# Business as usual? Psychological support at a distance

**DOI:** 10.1177/1359104520937378

**Published:** 2020-06-27

**Authors:** Lara Payne, Halina Flannery, Chandrika Kambakara Gedara, Xeni Daniilidi, Megan Hitchcock, Danielle Lambert, Charlotte Taylor, Deborah Christie

**Affiliations:** Child and Adolescent Psychology Service, University College London Hospital, London, UK

**Keywords:** E-therapies, remote therapies, telepsychology, telemental health, covid-19, psychological support, face-to-face therapies, paediatric psychology

## Abstract

The impact of COVID-19 has challenged the long accepted ‘norm’ in delivery of psychological therapy. Public policies designed to reduce transmission have made it extremely difficult to meet with service-users safely in the traditional face-to-face context. E-therapies have existed in theory and practice since technological progress has made them possible. They can offer a host of advantages over face-to-face equivalents, including improved access, greater flexibility for service-users and professionals, and cost savings. However, despite the emerging evidence and anticipated positive value, implementation has been slower than anticipated. Concerns have been raised by service-users, clinicians, and public health organisations, identifying significant barriers to the wide spread use of e-therapies. In the current climate, many clinicians are offering e-therapies for the first time, without prior arrangement or training, as the only viable option to continue to support their clients. This paper offers a clinically relevant review of the e-therapies literature, including effectiveness and acceptability dilemmas and challenges that need to be addressed to support the safe use and growth of e-therapies in psychology services. Further research is needed to better understand what might be lost and what gained in comparison to face-to-face therapy, and for which client groups and settings it might be most effective.

## Introduction

The impact of COVID-19 has challenged the long accepted ‘norm’ in delivery of psychological therapy. Shielding for those with underlying conditions, social distancing, and ‘lockdown’ designed to reduce transmission have made it extremely difficult to meet with service-users safely in the traditional face-to-face context. Remote working has meant finding alternative ways to offer psychological therapy.

E-therapies can be delivered by telephone, video-conferencing, and chat based interventions. Various terminologies are used to refer to e-therapies, including ‘telepsychology’, and ‘telemental health’, which may have developed from the use of telecommunication devices in some medical settings, referred to as ‘telemedicine’ or ‘telehealth’ (Hilty et al., 2013). We use ‘e-therapies’ in this paper to reflect the difference of our work to the medical model and to refer specifically to the delivery of psychological therapy. The majority of these are ‘synchronous’ requiring real time input from a clinician. We will not be discussing ‘asynchronous’ e-therapies which include email interactions, automated interventions, and self-help options.

E-therapies have existed in theory and in practice since technological progress has made them possible. The need for remote, online, digital options within psychological services is familiar, especially for those who cannot easily come to sessions in-person. E-therapies offer a host of advantages over face-to-face equivalents, including improved access, greater flexibility for service-users and professionals, and cost savings (Andersson & Titov, 2014). Systematic reviews and meta-analyses have shown promising outcomes for e-therapies in a range of populations (e.g. Barak et al., 2008; Tang et al., 2018). The NHS Long Term Plan, published before the COVID-19 outbreak, detailed hopes for increasing the availability of digital health care (NHS, 2019).

Despite the emerging evidence and anticipated positive value, implementation has been slower than anticipated (Barak et al., 2008; Wind et al., 2020). Concerns have been raised by service-users, clinicians, and public health organisations, identifying significant barriers to the wide spread use of e-therapies. Reservations focus on changes in the therapeutic relationship, reduced non-verbal interaction, maintaining confidentiality, responding appropriately to risk situations, and complying with the appropriate best practice guidelines (Barak et al., 2008). Local governance and practical issues (e.g. lack of adequate technology) have also prevented implementation. There also exists inequality for families and young people (e.g. homeless youth) that do not have access to technology or the environment to allow appropriate use.

These barriers have had to be rapidly addressed as e-therapies have become essential to the continued operation of psychological services. Recent guidance has encouraged increased flexibility and sensible decision making with regards to information governance, to enable previous barriers to be overcome and allow for continued patient care (Department of Health and Social Care, 2020; NHS, 2020). Consequently, many clinicians are offering e-therapies for the first time, without prior arrangement or training, as the only viable option to continue to support their clients. This paper offers a clinically relevant review of the e-therapies literature, including effectiveness and acceptability dilemmas and challenges, to support the continued use and growth of e-therapies in psychology services.

## E-therapies vs face-to-face: Effectiveness

### Adults

Perhaps the most notable step in the use of remote therapies in routine clinical practice in England was seen with the introduction of the Improving Access to Psychological Therapies (IAPT) initiative in 2008. IAPT began with the promise of over £170 million additional funding per annum to improve access to therapy for working age adults experiencing anxiety and depression (Clark et al., 2009). The model had ambitious targets, with two streams of treatment based on cognitive behavioural therapy; low intensity (for mild to moderate symptoms) and high intensity (for those with moderate to severe symptoms). Interventions included telephone-based guided self-help as well as internet-delivered programs for those experiencing mild to moderate symptoms of anxiety and depression. This allows increased accessibility, convenience and reduced therapist input, so a higher number of people can receive treatment at the same time (Thew, 2020).

This approach has been effective for mild to moderate anxiety and depression, but only if supported by a low intensity worker who checks in and reviews progress, offering further guidance and support. (Andersson & Cuijpers, 2009; Baumeister et al., 2014). There is increasing guidance on delivering ‘high intensity’ interventions online (Stott et al., 2013; Thew et al., 2019; Wild et al., 2016) and emerging evidence to suggest good outcomes for more severe problems. However, careful thought needs to be given to the practical considerations of offering e-therapies to clients experiencing more severe symptoms, including managing risk (Bower et al., 2013; Richards et al., 2018).

There is increasing evidence that e-therapies can be as effective as face-to-face treatments in adults for a range of presentations. Systematic reviews and meta-analyses have confirmed these findings for anxiety (Olthuis et al., 2016), psychiatric and somatic disorders (Carlbring et al., 2018), insomnia (Luik et al., 2017), substance abuse (Benavides-Vaello et al., 2013; Young, 2012), and depression (Andrews et al., 2018; Castro et al., 2020; Osenbach et al., 2013). A recent rapid evidence assessment of randomised controlled trials (RCTs) investigating synchronous e-therapies suggested that evidence is strongest for telephone and video-delivered interventions; Varker et al. (2019) revealed robust evidence for telephone and video-delivered interventions for adults with depression, anxiety, post-traumatic stress disorder, and adjustment disorder.

### Children and young people

There is some consensus amongst clinicians that e-therapies, especially video calling, may be particularly beneficial for working with children and young people either as the predominant mode of working, or possibly as an adjunct to face-to-face therapy (Richards & Simpson, 2015). In their meta-review of 21 reviews exploring the effectiveness of e-therapies for children and young people, Hollis et al. (2017) found that CBT was the most widely delivered and researched approach and that it was broadly effective. Meta-analyses comparing e-therapies to non-therapeutic (waitlist or placebo) controls revealed small-to moderate effects of computerised CBT on depression outcomes and moderate-to-large effects on anxiety outcomes (Hollis et al., 2017). Yet, Hollis and colleagues (2017) found larger effects of computerised CBT for adolescents and young adults compared to younger children, suggesting that some e-therapy interventions may be less effective for younger children. It is proposed that younger children may benefit from increased parental involvement to support engagement with e-therapies, particularly in the early stages of setting up an intervention (Pennant et al., 2015).

Further meta-analyses have concluded that e-therapies are comparable to face-to-face therapies for young people in terms of their impact on symptoms of anxiety and depression (Davies et al., 2014; Hollis et al., 2017; Ye et al., 2014). E-therapies have also been suggested to be highly acceptable to young people and their parents (Chakrabarti, 2015; Hollis et al., 2017), with qualitative feedback being broadly positive (e.g. Melnyk et al., 2015; Stasiak et al., 2014).

Research exploring non-CBT interventions is currently limited and uncertain. The relative minority of studies evaluating non-CBT interventions, such as computerised problem-solving therapy (Hoek et al., 2012) and attention/cognitive bias modification (Hollis et al., 2017; Pennant et al., 2015) are inconclusive due to low quality evidence. A solution-focused brief therapy intervention delivered to adolescents through one-to-one online chat was found to improve depression outcomes compared to waitlist controls and this was maintained over 4.5 months (Kramer et al., 2014). However, the impact on younger children is not explored and research in this area remains sparse.

Whilst findings appear promising for anxiety and depression, benefits remain unclear for young people with ADHD, autism, psychosis, PTSD, and eating disorders (Hollis et al., 2017). A Cochrane review of remotely delivered therapies designed to support children and young people with pain management also suggested minimal benefits of e-therapies in improving symptom severity, physical functioning, anxiety, and depression, though these conclusions were based on only small number of studies (Fisher et al., 2019).

## Client perspectives

### Adults

A substantial majority of adult studies suggest high acceptability and satisfaction (e.g. Bee et al., 2008; Turner, 2015; Simpson et al., 2001b). Service-users cite improved access and greater convenience as a significant advantage over comparable face-to-face therapy sessions, especially for those who face geographical, mobility, and financial limitations (Mohr et al., 2006; Simpson et al., 2001b). E-therapies are also more financially viable for clients than in-person alternatives (Bashshur et al., 2000; Cook & Doyle, 2002). A telepsychiatry service in a rural area of Canada found an estimated cost saving of $210 per client, based on the cost of travel and impact of attending in-person therapy on childcare and work commitments (Simpson et al., 2001a). High acceptability is also indicated by lower attrition rates in e-therapies when compared to a face-to-face equivalents (Mohr et al., 2008, 2012; Morland et al., 2004).

The remote nature of e-therapies may remove treatment barriers such as stigma around accessing psychological support, particularly for men, and could in turn increase disclosure in some contexts (Hilty et al., 2007; Turner et al., 2018). Some clients using telephone or text-based therapies have reported feeling able to talk more freely and feeling less concerned about how others may judge them (Turner, 2015). A recent adult survey suggested that 72% of adults would like to try digital psychotherapy, but when forced to choose between modalities, the majority preferred face-to-face (Renn et al., 2019).

### Children and young people

Young people are familiar with using the internet and digital devices as methods for communication; estimates from the UK and the US suggest that 47% of 5 to 10 year olds have a smartphone, increasing to 69% by the age of 12, and children aged 8 to 12 spend just under 5 hours using screens per day, increasing to over 7 hours per day for teenagers (Childwise, 2020; Common Sense, 2019). The privacy, flexibility, and control for the user that e-therapies offer have been suggested to be particularly valued by young people (Haig-Ferguson et al., 2010; Stallard et al., 2010; Sucala et al., 2012). Nevertheless, a significant majority of young people attending a Tier 3 CAMHS service reported a preference for face-to-face sessions (in clinic, at home, or at school) over e-therapies (Stallard et al., 2010).

## Clinician perspectives of e-therapies

Whilst e-therapies appear acceptable and effective, clinicians continue to have mixed views when asked to deliver them. E-therapies pose a new way of working with, and being with, clients. Some healthcare practitioners note ease of scheduling, more efficient use of clinical time, and reduced waiting times as potential advantages to e-therapies (Doze et al., 1999). They have also acknowledged the positive views of clients and potential for engagement described in the previous section, whilst holding onto concerns about the emotional and practical differences associated with e-therapies.

It is widely accepted that building a successful therapeutic relationship between therapist and client is more fundamental to the effectiveness of psychological interventions than the specific therapeutic model or approach (Haugh & Paul, 2008; Martin et al., 2000). Whilst there are different opinions as to what constitutes a ‘successful’ therapeutic relationship (Chadwick, 2006; Egan, 1990), the aspects of it that rely on human connection and building a subjective rapport may be compromised by e-therapies (Haig-Ferguson et al., 2019). Non-verbal communication (such as eye contact, facial expressions, and body language) is often cited as an important factor in the development of a therapeutic relationship and clinicians have raised concerns about the absence of such cues in telephone and text-based therapies, especially with regards to detecting emotions and gauging client engagement (McLaren et al., 1996).

However, studies investigating therapeutic relationship found adults and children receiving (predominantly CBT-based) e-therapies rated therapeutic alliance as high (Sucala et al., 2012). They further suggested that e-therapies are at least equivalent to face-to-face therapy in terms of therapeutic alliance (Cavanagh & Millings, 2013; Sucala et al., 2012). Agar (2020) extends this view and suggests that the technical cooperation required in e-therapies may even facilitate the development of therapeutic alliance by creating roles and goals in common.

## Practical and psychological considerations

There are a number of practical and psychological considerations that clinicians need address as they move to this new version of delivering therapy. These are discussed in more detail below. Box A describes a number of top tips and tricks that we have curated to help working remotely. These are not exhaustive.

**Box A.** TopTips for working remotely.

*Preparation* • If using video-conferencing software, ensure that it is sufficiently secure. Many of the available options recommend use of passwords (e.g. Zoom) to protect your video meetings. • Whichever method you choose, ensure that you are comfortable using the technology in advance. Practice using unfamiliar software with colleagues or friends first. • If possible, use a ‘waiting room’ so that you and any team members can get yourself prepared to welcome the client into the session. • Optimise your set-up such that you have a reliable internet connection with sufficient bandwidth, and a screen that is large enough to see your client’s facial expressions.
• Arrange a pre-therapy therapy call and have open discussions with clients about their thoughts and any concerns about e-therapies (especially if they have not chosen to access support in this way). • Offer practical advice and instructions to clients, especially if using video-conferencing software they have not used before. Consider developing a short guide with pictures which can be sent to them in advance of your first session. Be prepared to give a short tutorial to first time users. • When video-conferencing, keep your background neutral and consistent across sessions to help establish therapeutic boundaries. It may be helpful to recommend that clients also choose a consistent setting (e.g. room in their house) and make arrangements to ensure privacy during sessions (e.g. letting other household members know, turning off their phone). • Make appropriate ‘back up’ and risk management plans with clients to deal with technology failures or unexpected terminal of meetings to ensure their safety. This may include use of other modalities (e.g. telephoning them if video connection lost, or emailing them if telephone connection lost), and contacting their GP or next of kin if contact cannot be re-established. • If meeting with children and young people, ensure that appropriate parental supervision is available.
*During sessions* • Take time to begin well. Discuss confidentiality and do not assume someone is in a private space. Be explicit about how you are protecting the client’s confidentiality and privacy. • Set expectations and time boundaries. Some clients may prefer shorter sessions over video or telephone. • It might be necessary to re-contract the focus of psychological therapy that has transitioned from face-to-face to virtual. Some clients may not feel comfortable or safe when having sessions in their own homes. • When using telephone, be conscious of verbal competencies such as tone of voice, volume, and providing silence appropriately to help develop therapeutic relationship. • Discuss the option to turn off camera feedback on video calls, depending on client preference. • Be mindful of pace and the tendency for clients to disclose more in e-therapy sessions. Equally, be conscious of any personal desires to move faster or offer more to ‘compensate’ for the loss of the in-person experience for your clients. • Be prepared to be more active. With the absence of some non-verbal cues, it might be necessary to use more questions and be more directive. Consider how to demonstrate active listening if using the telephone. • Explain that sometimes things can get lost in communication (especially if it is on phone or email) but that we are always trying to convey warmth and understanding and that if the client is unsure how to ‘hear’ something we have said, to let us know. • Seek regular feedback. • Many video-conferencing options allow you to share your computer screen. This may be helpful to look at internet pages, worksheets or formulations together. Similarly, some programmes have a ‘whiteboard’ function which allows you to create a shared document. • Children and young people may wish to share their toys or use drawing materials during sessions. • Be creative and flexible. • Systemic working is possible. Many video-conferencing options will allow multiple participants and reflective teams can be used in different ways. • Take time to end well. Talk to clients about how they will take care and ground themselves after the session. *After sessions* • For many of us, offering e-therapies represents learning a new skill, and it is likely to be tiring at first. Take breaks between sessions, and consider reducing session length. • Be mindful of maintaining your own boundaries, especially if working from home and/or using personal equipment. • Ensure you have access to appropriate supervision when working remotely. Video supervision is common and generally effective (Pennington et al., 2003).

### Safety

Accessibility and geographical distance between therapist and client have led to concerns about liability and managing client safety. Assessing risk in a face-to-face context makes it possible to escort a client who expresses suicidal intent to A&E. Clients may disconnect from phone or video calls, requiring clinicians to rely on local teams and carers to ensure safety.

COVID-19 and social distancing make it more likely clinicians are working remotely and in isolation, creating an increased burden in managing risk without the benefit of a wider team in close proximity. It is important to ensure adequate supervision, support structures, and risk management protocols are in place when offering e-therapies.

### Confidentiality and setting

Since the 1990s, clinicians have recognised the threat to privacy that e-therapies may pose (Haas et al., 1996). It is difficult to create a safe, structured, neutral, and private environment when clients are speaking from home. Environmental distractors beyond clinician control may include household members, siblings/children, pets, excessive background noise which may all affect engagement with therapeutic content. Risk of eavesdropping means some clients may not find it possible to access a safe and private place in their own home to engage in e-therapy. This should be particularly considered if there are any safeguarding concerns both for young people and parents.

The potential for less formal environments may also mean e-therapies carry a greater risk of blurring professional boundaries. The clinician who dresses more casually may risk taking on a ‘buddy’ role and be viewed similarly as simply another friend whom the client talks with online (Andersen et al., 2001; Drum & Littleton, 2014). Delivering therapy from home can feel like an invasion of privacy for some clinicians (Haig-Ferguson et al., 2019); personal items and photographs visible during video calls may lead to a level of unintentional self-disclosure for both clients and clinicians that could interfere with boundaries and the therapeutic relationship. Some clients may also feel threatened by the clinician’s virtual presence in their home, which may challenge their sense of safety and impede therapeutic progress (Drum & Littleton, 2014).

### Reliance on technology

All clinicians offering e-therapies must be proficient in the technology they are using in order to instruct their clients and manage potential glitches that may arise e.g. with the software, equipment, or internet connection. Clinicians have highlighted the need for staff training on using the technology involved in effective therapy delivery (Haig-Ferguson et al., 2019). Questions have also been raised around the security of video-conferencing software, as they have the potential to be ‘hacked’, breaching confidentiality and information governance policies (e.g. Paul, 2020). All e-therapies must ensure that the use and storage of data meet relevant healthcare standards for handling patient data.

### Seeing oneself on screen

Video-conferencing provide users with feedback from their own camera. As seeing oneself is not the norm in face-to-face communication it can be distracting during video conversations unless it is switched off. Visual feedback increases self-awareness in a video chat context, affecting resulting communication (Miller et al., 2017). For individuals with social anxiety, where self-focus is a central maintaining factor seeing oneself may derail therapeutic benefit (Clark & Wells, 1995). Alternatively, clients may experience an increased sense of ‘togetherness’ seeing themselves alongside their therapist on screen, and this may encourage valuable transference from an early stage (Agar, 2020).

### One size does not fit all

Reliance of e-therapies on technology may constitute a barrier for anyone who cannot afford access to such devices and/or the internet; there remains a significant gap in laptop and smartphone ownership amongst young people from low and high income homes (Common Sense, 2019). Similarly, it may not be feasible for those who do not have the space or privacy to engage in e-therapies in their home. Younger children, clients with learning disabilities, ADHD, or hearing or visual problems, and older adults who are less familiar with technology, may all find it more difficult to access the benefits of e-therapies. However, the flexible and remote nature of e-therapies may offer a more viable option to client populations who have historically fallen between gaps or struggled to engage with traditional psychology service provision; for example, it has been suggested that the internet may provide a more comfortable medium of communication for people with autism (Benford & Standen, 2009).

The majority of e-therapies research has focused on structured, protocol-driven therapies such as computerised CBT. Clinicians have raised concerns about e-therapies services offering “production line therapy” (Turner et al., 2018) that fit with a CBT-based model but may reduce the opportunities to access alternative therapies that could better meet client needs (Wesson & Gould, 2010).

## Group e-therapies

Evidence-based group therapies are a popular therapeutic approach applied for a range of psychological distresses in adult, adolescent, and child populations (e.g. Essau et al., 2012; Nardi et al., 2016; Stallard et al., 2008). Group interventions have been demonstrated to be as effective as one-to-one therapy in improving social interaction and learning (Haight & Gibson, 2005) and overcoming stigma in accessing mental health services (Weisz et al., 2006) whilst remaining considerably more economical (Vos et al., 2005). Consequently, it is perhaps not surprising that mental health services are keen to use and promote group approaches (Lorentzen & Ruud, 2014). Video-based ‘real time’ online group therapies may be an advantageous way to increase accessibility, uptake and improve retention rates (Christensen & Hickie, 2010).

A systematic review of 17 adult studies exploring group therapy offered via video-conferencing indicated a ‘trend’ towards clinical effectiveness with improvements in health, mental health, and self-efficacy outcomes (Banbury et al., 2018) comparable to in-person therapeutic groups (Adamski & Alfaro, 2009; Khatri et al., 2014). Overall feasibility and accessibility of group e-therapies was also found to be high (Banbury et al., 2018). Whilst some participants expressed a preference to access in-person groups, this preference was outweighed by the perceived benefit of the convenience of online groups (Lopez et al., 2020).

The literature is mixed in evaluating the group therapy process online. Virtual group therapy creates the issue of the ‘disembodied group’, where group members cannot easily respond to cues such as the use of gaze as prompts to talk. Some studies have suggested that virtual groups are able to replicate group processes such as bonding (Banbury et al., 2018), whilst other studies offering anger management and DBT found participants feel less connected (Lopez et al., 2020), and exhibit lower alliance with the group facilitator than participants in face-to-face groups (Greene et al., 2010). However, online groups and improved access may also lead to the strengthening of other therapeutic factors. Weinberg (2020) argues that expanding beyond the boundaries of the physical world may offer a more powerful experience, and the chance to include greater societal and cultural diversity in online groups strengthens universality and existential factors to good therapeutic effect.

Strong therapeutic alliance, working alliance, and group cohesion are also critical factors for therapy engagement and positive outcomes when working with young people (Hawley & Garland, 2008; Shirk & Karver, 2003). Recapture Life, a RCT for adolescents and young adults following cancer treatment, compared an online video-based CBT group with a peer support group and waiting list control. Participants in the CBT group rated strong therapeutic alliance with their therapist from the start of intervention which was sustained over the treatment programme (McGill et al., 2017). Results suggest that it may be possible for interpersonal processes, such as working alliance between participant and therapist and group cohension, to be developed through online group delivery as scores were similar or higher when compared to face-to-face interventions with young people and adults (McGill et al., 2017).

Overall, e-groups may be a viable alternative to in-person groups but further research is needed to understand the processes by which they effect change. There is a suggestion that therapeutic processes could operate over video but facilitators of virtual groups may need to take additional measures and time to support this.

## Conclusions and future directions

Methodological limitations mean it is challenging to draw firm conclusions about the efficacy of e-therapies in both adults and young people. Heterogeneous delivery platforms have often been grouped and analysed together, ignoring key distinctions between synchronous and asynchronous modalities as well as amount of therapeutic input (Osenbach et al., 2013). The degree of the therapist involvement ranges on a continuum from automated reminders and emails, to regular real time telephone or video calls in a similar model to face-to-face sessions (Shafran et al., 2009). Individual clinicians may use any combination of these methods. It seems imperative, therefore, to explore differential evidence supporting each of these modes of delivery for different diagnoses and other population characteristics in order to understand whether the outcome is related to the intervention itself, the modality of e-therapies or their interaction for specific client populations (Hollis et al., 2017).

Ways of working when face-to-face with clients in a therapy room may feel difficult to translate through a computer screen, for example, when using play-based or physical interventions such as narrative sandplay (Lee, 2018). Despite these challenges, the authors have been experimenting with ways to deliver approaches involving activities that might at first feel easy to lose in translation; for example, in continuing to use beads to create narratives about people’s lives using a Beads of Life approach (Portnoy et al., 2016), and running online groups for young people with cognitive impairment. This has meant being creative and at times courageous.

The advent of the COVID-19 pandemic has thrust the fields of clinical psychology and psychotherapy firmly into the digital age and necessitated the use of technology to continue our work. A growing evidence base and high acceptability suggests that e-therapies could continue to occupy a unique and valuable role in psychology services. Whilst we traverse this new territory it is imperative that we evaluate the effectiveness, accessibility and acceptability of e-therapies by seeking feedback from both clients and clinicians on the experience and the outcomes of therapy. Anecdotally, many of us are finding this way of working exhausting. It is unclear whether it is exhausting simply because it represents a new way of working, or because we are straining to adapt to relating to our clients differently and working with a more limited range of non-verbal communication.

In the midst of crisis, there is an opportunity to contribute practice-based evidence to the existing body of RCTs and manualised e-therapy research. There is a need to explore effectiveness with complexity, different therapeutic approaches, and contexts. For instance, little attention has been paid to the role of e-therapies in working with families and wider systems. Further research is needed to better understand what might be lost and what gained in comparison to face-to-face therapy, and for which client groups and settings it might be most effective. E-therapies are a good adjunct, and in the current climate are helping us deliver business as near to usual as possible, but we would caution the belief that it can overtake face-to-face encounters in the future when we are ‘back to normal’.
